# Antibacterial Performance of Defect-Enriched PtPdCuFe High-Entropy Alloy Nanozymes

**DOI:** 10.3390/molecules31193506

**Published:** 2026-10-01

**Authors:** Jiawei Zhang, Zhiyuan Li, Weng-Chon (Max) Cheong, Awanisa Wubuli, Sitraka Ny Aina Raharivelo, Ziheng Chen

**Affiliations:** 1 Macao Institute of Materials Science and Engineering (MIMSE), Macau University of Science and Technology, Macao 999078, China; 2 School of Chinese Materia Medica, China Pharmaceutical University, Nanjing 211198, China; 3 State Key Laboratory of Climate System Prediction and Risk Management/Key Laboratory of Meteorological Disaster, Ministry of Education/Collaborative Innovation Center on Forecast and Evaluation of Meteorological Disasters, Nanjing University of Information Science and Technology, Nanjing 210044, China; 202451010005@nuist.edu.cn; 4 School of Civil Engineering and Architecture, Anhui University of Science & Technology, Huainan 232001, China

**Keywords:** nanozyme, structural defects, antibacterial materials, high-entropy

## Abstract

Antibiotic resistance limits infected-wound treatment and motivates the search for antibiotic-independent antibacterial materials. Here we report monodisperse PtPdCuFe high-entropy alloy (HEA) nanozymes with multi-enzyme activities, 2.14 ± 0.18 nm in diameter, prepared by oleylamine-assisted pyrolysis. We propose that synergistic metal interactions and abundant defects may endow the nanozymes with pH-gated peroxidase-like (POD-like), catalase-like (CAT-like), and superoxide dismutase-like (SOD-like) activities. At pH 5.5, the nanozymes exert POD-like activity to decompose H_2_O_2_ into hydroxyl radicals (•OH), triggering an ROS burst that destroys the bacterial wall, causes intracellular content leakage, and ultimately kills bacteria. At pH 7.4, they exert SOD- and CAT-like activities: ~84% of H_2_O_2_ was removed within 25 min and O_2_ rose to ~50% within 60 s, indicating potential to alleviate wound hypoxia, yet to be validated in future animal experiments. At the lowest effective concentration tested (16 µg·mL^−1^), bactericidal efficiencies against *Escherichia coli* and *Staphylococcus aureus* were ~56% and ~57%, respectively. At 64 µg·mL^−1^, killing reached ~90%, with >90% of bacteria showing severe membrane disruption and protein leakage. At this concentration, ~90% of mammalian cells remained viable and the hemolysis rate stayed below 5%.

## 1. Introduction

Bacterial wound infections remain difficult to manage [1,2,3,4], and decades of antibiotic misuse have accelerated the emergence of multidrug-resistant bacteria [5]. Conventional antibiotic therapies are often limited by increasing antimicrobial resistance, restricted bioavailability, adverse effects, and the complexity of the infection-associated microenvironment [6,7,8]. Moreover, the early stage of wound infection is typically characterized by local acidification, hypoxia, abnormal levels of reactive oxygen species (ROS), and persistent release of inflammatory mediators. These changes make it difficult for conventional antibacterial strategies to simultaneously achieve efficient bacterial eradication, minimize the development of drug resistance, and regulate the complex pathological microenvironment [9,10,11]. Therefore, the development of novel antibacterial materials with good biosafety and the capacity to respond to changes in the infection microenvironment has become an active area of nanomedicine [12].

Advances in nanotechnology have provided new opportunities to overcome the limitations of conventional antimicrobial agents [13,14]. Among various nanomaterials, nanozymes have attracted considerable attention in antibacterial therapy because they combine enzyme-mimicking catalytic activities with the stability, tunability, and scalable synthesis of nanomaterials [15,16,17]. Unlike antibiotics that rely on specific biological targets, nanozymes with peroxidase-like (POD-like) or oxidase-like (OXD-like) activities can convert hydrogen peroxide or molecular oxygen into ROS such as hydroxyl radicals (•OH) and superoxide anion radicals (•O_2_^−^) [18,19]. These ROS can induce lipid peroxidation, disrupt bacterial membrane integrity, and cause leakage of intracellular components, including proteins and nucleic acids. In addition, ROS-induced perturbation of bacterial redox homeostasis results in oxidative damage and may reduce the likelihood of resistance arising from mutations in a single molecular target [20,21,22,23,24,25]. Accordingly, multimetallic-based [26], metal oxide [27], carbon-based [28], and single-atom [29] nanozymes have been extensively investigated for antibacterial applications and have demonstrated considerable potential for bacterial eradication and biofilm elimination. Although elevated ROS levels facilitate rapid bacterial killing during the early stage of wound infection, persistent ROS accumulation may damage normal cells and delay tissue repair [30,31]. An ideal antibacterial nanozyme should therefore be capable of dynamically regulating ROS generation, multi-enzyme activities and scavenging according to changes in the pathological microenvironment [32]. During the early stage of wound infection, the mildly acidic microenvironment may activate POD/OXD-like catalytic pathways and enhance oxidative antibacterial activity. As the wound returns to a neutral or mildly alkaline state during recovery, the catalytic behavior may switch to catalase-like (CAT-like) and superoxide dismutase-like (SOD-like) activities that reduce excessive oxidative stress and alleviate local hypoxia, shifting the material from killing bacteria to restoring a microenvironment that supports repair [33,34,35,36,37]. Recent advances in pH-responsive and multienzyme-cascade nanozymes suggest that such dynamic catalytic regulation may provide an effective strategy for improving anti-infective therapy. Achieving this adaptive regulation imposes stringent requirements on nanoparticle composition, atomic arrangement, electronic configuration, and defect structure, for which high-entropy materials provide an attractive platform.

With the rapid development of high-entropy materials, their definition has expanded from systems containing five or more metallic elements to include certain quaternary systems [38]. The four characteristic high-entropy effects—cocktail, lattice distortion, sluggish diffusion, and configurational entropy—provide compositional tunability, abundant catalytic active sites, and structural stability that are difficult to achieve simultaneously in conventional materials [39,40]. High-entropy materials therefore offer a promising strategy for constructing environment-responsive and multienzyme-mimicking antibacterial nanozymes. For example, Han et al. [41] reported a PtFeCuCoNi HEA nanozyme exhibiting pH-gated sequential redox biocatalysis. In the acidic microenvironment of infected wounds, the nanozyme exhibited POD/OXD-like activity and generated ROS, enabling the elimination of methicillin-resistant *Staphylococcus aureus* (MRSA) and disruption of bacterial biofilms at a low concentration. Similarly, Li et al. [42] demonstrated that a CoNiCuZnCe high-entropy nanozyme combined POD-like catalytic activity with near-infrared photothermal properties. The nanozyme decomposed H_2_O_2_ to •OH and exhibited efficient photothermal conversion under 808 nm laser irradiation. When incorporated together with methylene blue into chitosan-based hydrogel microneedles, the system integrated nanozyme-mediated •OH generation, methylene-blue-mediated photodynamic production of singlet oxygen, and photothermal effects, resulting in efficient eradication of drug-resistant Listeria monocytogenes with strong bactericidal efficiency. Despite these advances, the preparation of ultrasmall, monodisperse, multi-enzyme activity, defect-rich high-entropy nanocrystals remains challenging, and the structure–activity relationships among atomic-scale defects, pH-gated multienzyme-like activities, and antibacterial effects remain poorly defined. High-entropy materials nevertheless hold considerable potential for the development of adaptive antibacterial systems for infected wounds.

In the present study, multi-enzyme activities PtPdCuFe HEA nanozymes with an average particle size of 2.14 ± 0.18 nm were synthesized via an oleylamine-assisted pyrolysis method. The resulting nanoparticles exhibited an fcc-dominated solid-solution structure enriched with defects, including twins and grain boundaries. We hypothesize that the defect-rich structure, together with multimetallic synergistic interactions, provides the structural basis for the pH-gated POD-, CAT-, and SOD-like activities of PtPdCuFe nanozymes [43]. Under acidic infection conditions, the nanozymes generate •OH that causes bacterial wall rupture, intracellular content leakage, and ultimately bacterial death, whereas under neutral conditions associated with wound recovery, they remove excess ROS and catalyze H_2_O_2_ decomposition to produce O_2_. Whether these properties ultimately promote wound healing requires further validation in animal models. By integrating aberration-corrected electron microscopy, spectroscopic analysis, in vitro antibacterial and biocompatibility evaluations, and bacterial transcriptomic analysis, this study investigates the relationships among the structure of PtPdCuFe HEA nanozymes, their multienzyme-like catalytic properties, bacterial membrane damage, and metabolic dysregulation, and aims to establish a preliminary structure–activity relationship linking material defects, multienzyme catalysis, and antibacterial activity, thereby expanding the range of candidate nanomaterials for the treatment of infected wounds.

## 2. Results

### 2.1. Structural Characterization of the High-Entropy Nanozyme

**Figure 1:**
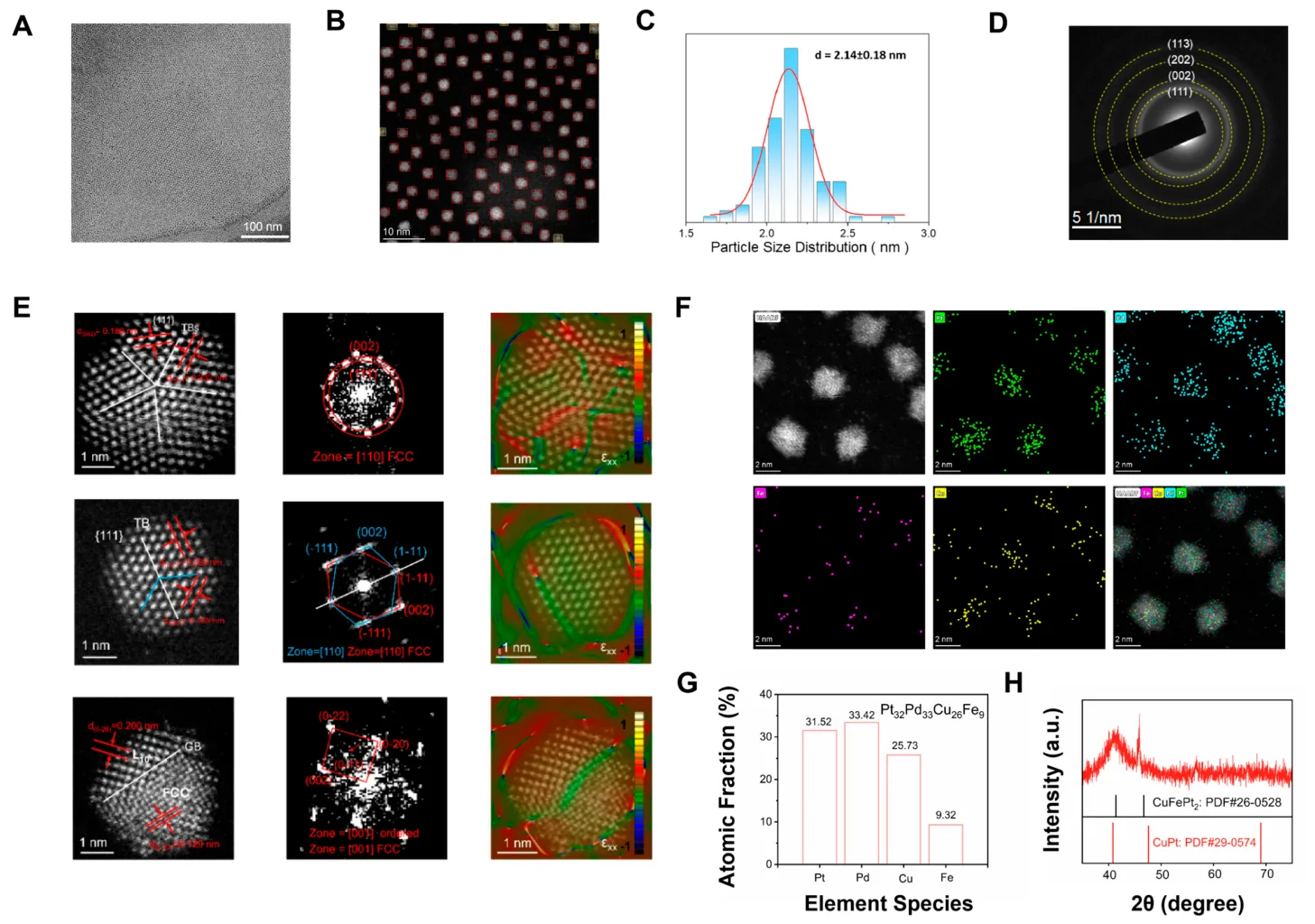
Structural characterization of PtPdCuFe high-entropy alloy nanozymes. (**A**) TEM image of PtPdCuFe nanozymes. (**B**) Aberration-corrected HAADF-STEM image of PtPdCuFe nanozymes. (**C**) Particle-size distribution of PtPdCuFe nanozymes. (**D**) SAED pattern of PtPdCuFe nanozymes. (**E**) Single-particle GPA analysis of PtPdCuFe nanozymes. (**F**) EDS elemental mapping of PtPdCuFe nanozymes. (**G**) ICP-MS quantitative analysis of the elemental composition of PtPdCuFe nanozymes. (**H**) XRD pattern of PtPdCuFe nanozymes. Experiments were repeated independently (**A**,**B**,**F**) three times with similar results.

PtPdCuFe HEA nanozymes were successfully synthesized by oleylamine-assisted pyrolysis following synthetic process screening (Figure S9). Transmission electron microscopy (TEM) revealed well-dispersed, spherical nanoparticles with ultrasmall dimensions (Figure 1A). Aberration-corrected high-angle annular dark-field scanning transmission electron microscopy (HAADF-STEM) was subsequently employed to characterize the morphology and particle-size distribution. The PtPdCuFe nanoparticles exhibited excellent monodispersity and a uniform spherical morphology (Figure 1B), with a mean diameter of 2.14 ± 0.18 nm (Figure 1C). Sharp diffraction peaks were observed in the X-ray diffraction (XRD) pattern, indicating the crystalline nature of the synthesized HEA nanozymes (Figure 1H). Selected-area electron diffraction (SAED) analysis revealed clear and continuous diffraction rings (Figure 1D), indicating that the samples consisted of numerous randomly oriented crystalline nanoparticles. Radial integration of the diffraction rings yielded an intensity profile (Figure S1), in which the diffraction signals could be assigned to the characteristic (111), (002), (202), and (113) planes of an fcc structure, confirming the predominantly fcc crystalline structure of the synthesized nanozymes.

To elucidate their atomic-scale structural characteristics, representative PtPdCuFe nanoparticles were examined using aberration-corrected HAADF-STEM, fast Fourier transform (FFT) analysis, interplanar-spacing measurements, and geometric phase analysis (GPA). Three representative microstructural configurations were identified (Figure 1E): fivefold-twinned nanoparticles, nanoparticles containing twin boundaries, and nanoparticles exhibiting local atomic ordering. These observations indicate that the PtPdCuFe nanoparticles do not consist of structurally perfect fcc crystals but instead contain complex local atomic arrangements, a characteristic commonly observed in high-entropy alloy nanomaterials. The first representative nanoparticle exhibited a characteristic fivefold-twinned structure. Multiple crystalline domains were clearly distinguished in the aberration-corrected STEM image, and the corresponding lattice fringes were assigned to fcc-related crystallographic planes. The measured interplanar spacings were approximately 0.232 nm for the (111) plane and 0.186 nm for the (002) plane (Figure S2). The corresponding FFT pattern further confirmed the fcc structure along the [110] zone axis, with the indexed diffraction spots matching the lattice planes marked in the STEM image. Line-intensity profiles exhibited regularly spaced periodic atomic peaks, allowing accurate determination of the interplanar distances. Fivefold-twinned structures are generally associated with the accumulation of internal strain because the geometric mismatch between adjacent fcc domains cannot be completely relaxed at the nanoscale. The second representative nanoparticle contained a distinct twin boundary. Atomic-resolution STEM images showed two fcc domains connected through a twin interface, with {111} lattice planes extending across the particle. The measured interplanar spacings were approximately 0.228 nm for the (111) plane and 0.189 nm for the (002) plane, which were comparable to those observed in the fivefold-twinned nanoparticles (Figure S3). The corresponding FFT pattern contained two sets of fcc diffraction spots originating from two twinned crystalline domains coexisting along the [110] direction. Direct visualization of the twin boundary provided clear evidence of local structural heterogeneity within the PtPdCuFe HEA nanoparticles. The third representative nanoparticle exhibited more complex local atomic ordering. Both fcc regions and partially ordered domains could be observed in the STEM image. The measured interplanar spacings were approximately 0.200 nm for the (020) plane and 0.129 nm for the (022) plane (Figure S4). The FFT pattern simultaneously contained diffraction signals corresponding to the fcc phase and ordered domains, indicating the presence of local atomic ordering within the HEA nanoparticles. GPA was used to visualize the local strain distribution within individual nanoparticles. As shown in the strain maps, the strain field was highly heterogeneous. Pronounced strain accumulation was observed at twin boundaries, grain-boundary-like interfaces, and interfaces between fcc regions and locally ordered domains, with distinct tensile and compressive strain regions. These observations suggest that lattice distortion is substantially amplified at structural defects and interfacial regions. Such strain heterogeneity may arise from the combined contributions of multimetallic atomic configurations, twin formation, and short-range ordered regions. Collectively, aberration-corrected HAADF-STEM imaging, FFT analysis, interplanar-spacing measurements, and GPA demonstrated that PtPdCuFe HEA nanoparticles predominantly possess an fcc crystalline structure while containing abundant and complex local structural defects, including fivefold twins, twin boundaries, grain-boundary-like interfaces, locally ordered atomic domains, and heterogeneous strain fields. These atomic-scale structural characteristics may be associated with catalytic performance. Twin boundaries, lattice distortion, and local strain fields may regulate the surface electronic structure of the nanoparticles, alter the adsorption energies of reaction intermediates, and provide additional catalytically active sites, thereby contributing to the catalytic activity and stability of PtPdCuFe HEA nanozymes, although the contribution of individual defect types to catalysis has not been isolated.

The elemental distributions of Pt, Pd, Cu, and Fe were investigated by energy-dispersive X-ray spectroscopy (EDS) coupled with aberration-corrected HAADF-STEM. All four elements were uniformly distributed within individual nanoparticles, with no obvious elemental segregation or enrichment. These results indicate effective mixing of the four metallic components and the formation of a homogeneous multicomponent alloy structure. Inductively coupled plasma mass spectrometry (ICP-MS) revealed atomic percentages of 31.52%, 33.42%, 25.73%, and 9.32% for Pt, Pd, Cu, and Fe, respectively, indicating that all four components were present as major constituents of the PtPdCuFe HEA nanoparticles (Figure 1G). Taken together, these findings demonstrate the synthesis of ultrasmall, highly monodisperse, and defect-rich PtPdCuFe HEA nanozymes [44,45,46].

### 2.2. Enzyme-Mimicking Activities and Colloidal Stability of PtPdCuFe HEA Nanozymes

**Figure 2:**
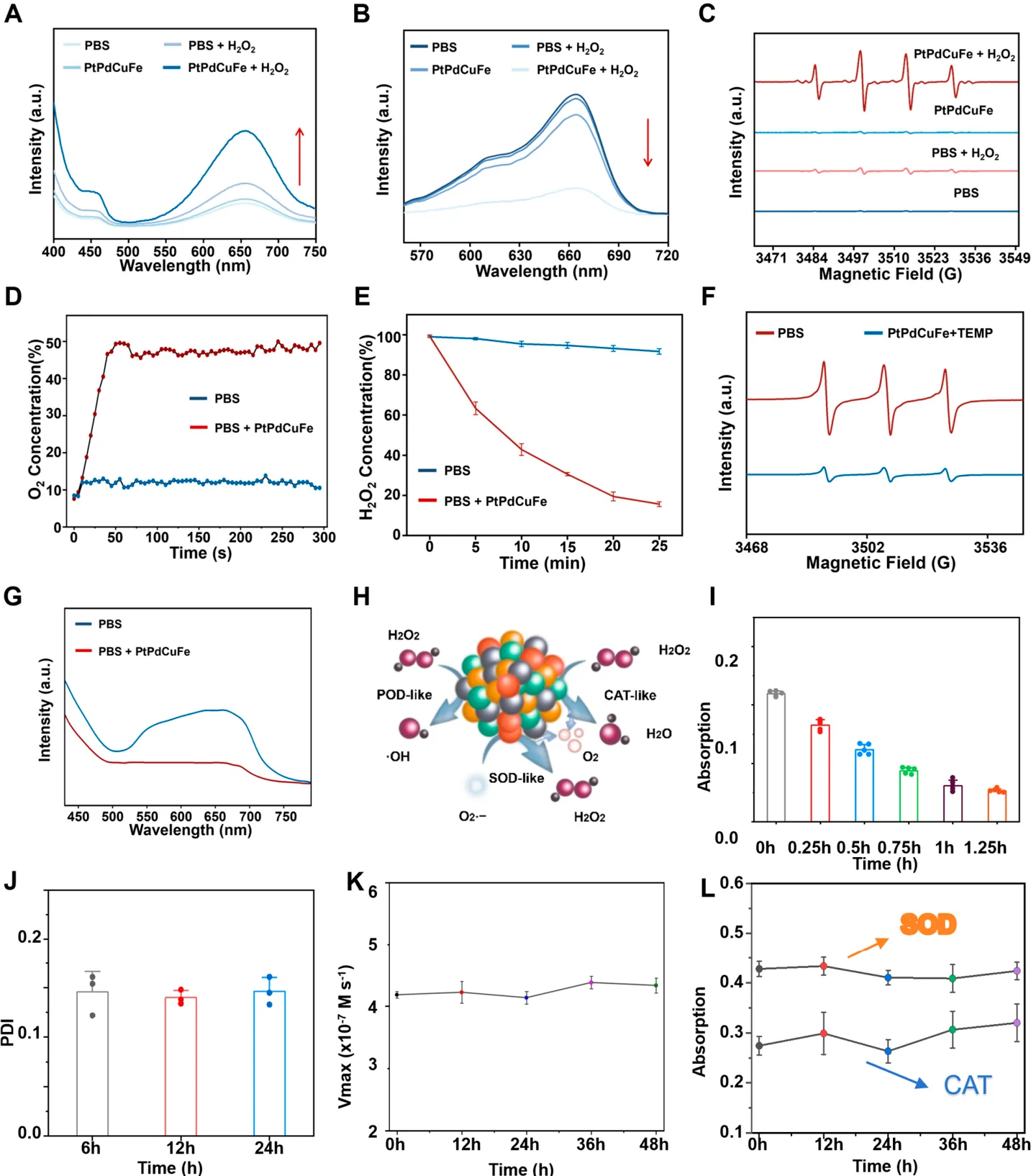
Enzyme-mimicking activities and stability of PtPdCuFe HEA nanozymes. (**A**) TMB oxidation under acidic conditions in different experimental groups. (**B**) MB degradation under acidic conditions. (**C**) EPR detection of •OH under acidic conditions. (**D**) O_2_ concentration under neutral conditions. (**E**) H_2_O_2_ consumption under neutral conditions. (**F**) EPR evaluation of singlet-oxygen (^1^O_2_) scavenging under neutral conditions. (**G**) Detection of •O_2_^−^ using the NBT assay. (**H**) Schematic illustration of the triple enzyme-mimicking catalytic activities of PtPdCuFe NPs. (**I**) OD values for •O_2_^−^ detection using NBT as the substrate under neutral conditions. (**J**) PDI of PtPdCuFe NPs at different time points. (**K**) Stability of POD-like activity at pH 5.5 over time. (**L**) Stability of CAT- and SOD-like activities at pH 7.4 over time. Experiments were repeated independently (**A**–**G**,**I**–**L**) three times with similar results.

To systematically evaluate the catalytic multifunctionality of PtPdCuFe high-entropy nanozymes, this study investigated their POD-like, CAT-like, and SOD-like activities under acidic conditions mimicking bacterial infection (pH 5.5) and under neutral physiological conditions (pH 7.4). In addition to these structural defects, the random arrangement of Pt, Pd, Cu, and Fe within the lattice creates a wide range of local coordination environments. We reasoned that this compositional disorder would provide a correspondingly wide range of active sites, and therefore a catalytic response that varies with pH. The POD-like activity of PtPdCuFe nanoparticles (NPs) under mildly acidic conditions was first evaluated using 3,3′,5,5′-tetramethylbenzidine (TMB) as the chromogenic substrate in the presence of H_2_O_2_ (Figure 2A). Upon addition of the nanozyme at pH 5.5, the reaction solution rapidly turned blue and exhibited a pronounced absorption peak at 652 nm, indicating conversion of TMB into its oxidized form (oxTMB). A methylene blue (MB) degradation assay confirmed the oxidative catalytic capability of the system, indicating the formation of strongly oxidizing species (Figure 2B). To identify the ROS generated, electron paramagnetic resonance (EPR) spectroscopy was performed using 5,5-dimethyl-1-pyrroline N-oxide (DMPO) as a spin-trapping agent. A characteristic 1:2:2:1 quartet signal was observed, confirming the generation of •OH (Figure 2C). These findings demonstrate that, under acidic conditions mimicking the microenvironment of infected wounds, PtPdCuFe nanozymes catalyze H_2_O_2_ decomposition through POD-like activity to generate bactericidal •OH, thereby enhancing ROS-mediated antibacterial activity. In contrast to the pro-oxidative POD-like activity observed under acidic conditions, CAT-like activity was markedly enhanced at neutral pH. Dissolved oxygen measurements showed that the O_2_ concentration increased rapidly and continuously after addition of PtPdCuFe NPs at pH 7.4, whereas no such increase occurred in the control (Figure 2D). H_2_O_2_ consumption experiments demonstrated efficient removal of exogenous H_2_O_2_ by the nanozyme (Figure 2E). This CAT-like behavior may be beneficial in two ways: removal of cytotoxic H_2_O_2_ reduces oxidative stress, and the resulting O_2_ alleviates local hypoxia and creates a more oxygenated micro-environment, indicating its potential in promoting wound repair. SOD-like activity was evaluated using the nitroblue tetrazolium (NBT) photoreduction assay (Figure 2G). At pH 7.4, PtPdCuFe NPs inhibited NBT reduction, indicating effective scavenging of •O_2_^−^ (Figure 2I). The nanozyme also exhibited scavenging activity toward singlet oxygen under neutral conditions (Figure 2F).

To evaluate the long-term stability of the catalytic system, the polydispersity index (PDI) of the nanoparticles was measured at different time points (Figure 2J). PtPdCuFe NPs maintained a stable, narrow size distribution over 24 h. Catalytic stability was subsequently examined at pH 5.5 and pH 7.4 after different storage intervals (Figure 2K,L). The nanoparticles retained essentially unchanged catalytic activity for up to 48 h.

Collectively, these results demonstrate a clear pH-dependent switch in the dominant catalytic pathways of PtPdCuFe nanozymes. Under acidic conditions, POD-like activity predominates and promotes the generation of •OH for bacterial killing. Under neutral conditions, CAT- and SOD-like activities become dominant, facilitating the removal of H_2_O_2_ and •O_2_^−^ while continuously generating O_2_ [47,48,49,50,51,52]. Based on our current experimental observations, we speculate that this pH responsiveness may originate from the synergistic effects among multiple metallic elements and the defect-rich surface distribution of the PtPdCuFe HEA nanozymes. Such pH-gated redox biocatalysis may enable efficient bacterial elimination in acidic infection environments while reducing excessive oxidative stress under neutral conditions. We will further investigate the underlying mechanism of this pH-dependent enzyme-mimicking activity through theoretical calculations in our future work.

### 2.3. Biosafety and Antibacterial Performance of PtPdCuFe HEA Nanozymes

Having established the pH-responsive multienzyme-mimicking activities of PtPdCuFe HEA NPs, we evaluated their biocompatibility and antibacterial performance using colony counting, Live/Dead fluorescence staining, hemolysis assays [53].

**Figure 3:**
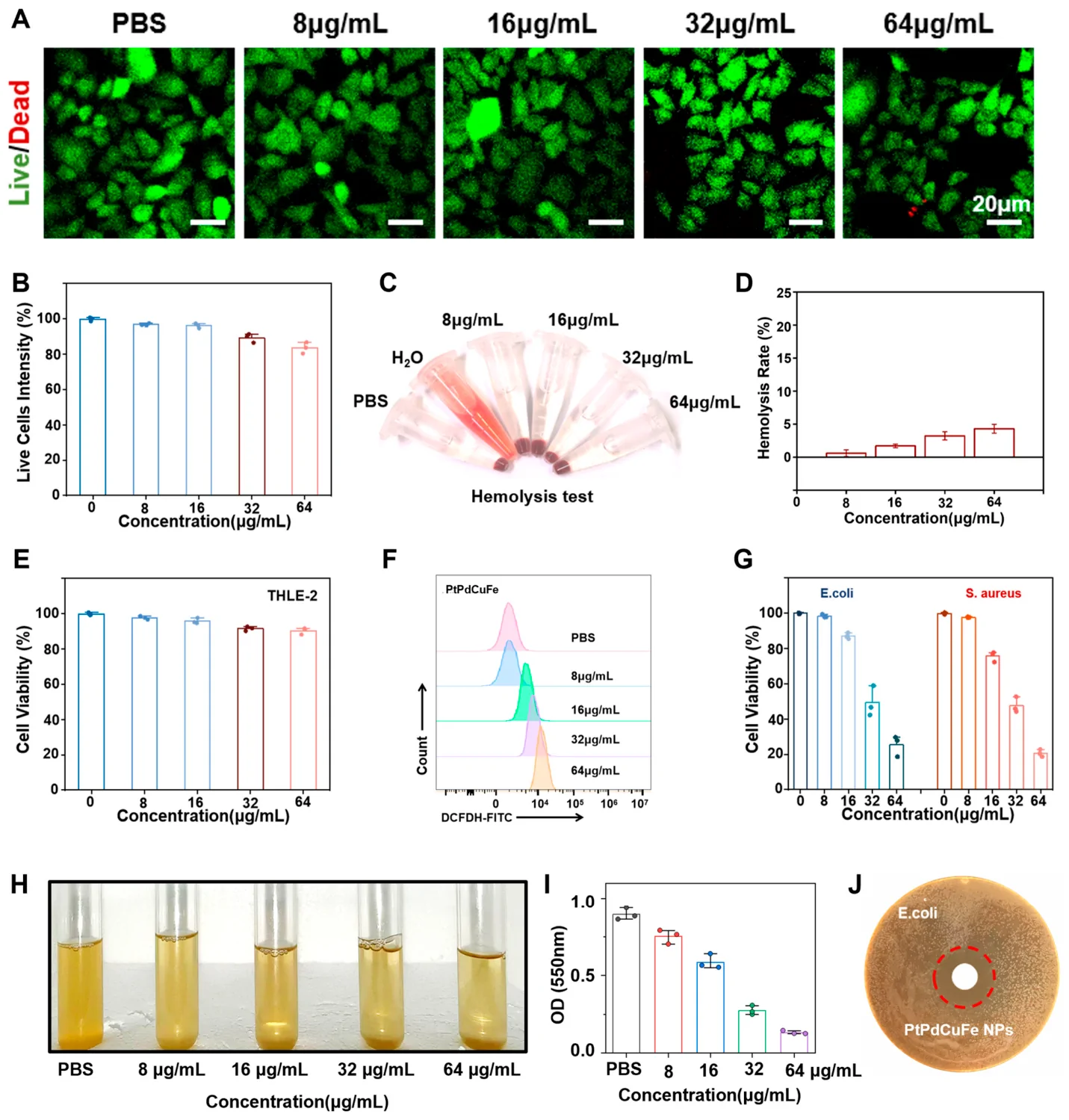
Biosafety and antibacterial performance of PtPdCuFe HEA nanozymes. (**A**) Live/Dead fluorescence images of normal cells treated with different concentrations of PtPdCuFe NPs. (**B**) Quantitative analysis of viable cells following treatment with different concentrations of PtPdCuFe NPs. (**C**) Hemolysis assay of PtPdCuFe NPs at different concentrations. (**D**) Quantitative hemolysis rates. (**E**) Cytotoxicity of PtPdCuFe NPs toward normal cells at different concentrations. (**F**) Flow-cytometric analysis of ROS levels following treatment with different concentrations of PtPdCuFe NPs. (**G**) Antibacterial activity of PtPdCuFe NPs against *S. aureus* and *E. coli*. (**H**) Broth turbidity assay. (**I**) Colorimetric analysis of bacterial turbidity. (**J**) Agar disk-diffusion assay of PtPdCuFe NPs. Experiments were repeated independently (**A**–**J**) three times with similar results.

PtPdCuFe NPs were first co-incubated with THLE-2 cells at concentrations ranging from 0 to 64 μg mL^−1^, and cell viability was assessed using Live/Dead staining. Fluorescence images showed predominantly green fluorescence corresponding to viable cells at all tested concentrations, with negligible red fluorescence from dead cells (Figure 3A,B). Quantitative analysis revealed no substantial reduction in cell viability across the tested concentration range. It should be noted, however, that metal ion leaching under physiological conditions may also exert a non-negligible influence on cell viability. Owing to limitations in experimental conditions, the present study did not investigate this aspect, and systematic evaluation of ion leaching and its associated cytotoxicity will be addressed in our future work. Hemolysis experiments showed that hemolysis rates remained below 5% at all tested concentrations (Figure 3C,D). Viability in the MTT assay remained above 90% at 64 μg mL^−1^ (Figure 3E). Intracellular ROS levels in bacteria following PtPdCuFe NP treatment were then determined using the ROS-sensitive probe DCFH-DA. Intracellular ROS oxidize DCFH to highly fluorescent dichlorofluorescein (DCF). As shown in Figure 3F, in the presence of H_2_O_2_, bacterial cells treated with 16 μg mL^−1^ PtPdCuFe NPs exhibited substantially enhanced DCF fluorescence compared with the control group, indicating pronounced intracellular ROS accumulation. This observation was consistent with the Fenton-like •OH-generating activity identified under acidic conditions and supports an ROS-mediated antibacterial mechanism involving oxidative damage to bacterial membranes and bio-molecules [54]. The antibacterial activity of PtPdCuFe NPs was quantitatively evaluated using standard colony-counting assays with Gram-positive *Staphylococcus aureus* and Gram-negative *Escherichia coli* as model strains. Using the MTT assay to evaluate bacterial viability, we found that PtPdCuFe NPs exhibited antibacterial activity in the presence of H_2_O_2_, with a bacterial survival rate of approximately 80% at 64 μg mL^−1^ (Figure 3G). The concentration-dependent inhibition of bacterial growth was supported by broth turbidity measurements (Figure 3H), which yielded an MBC/MIC ratio of 2, indicative of the bactericidal activity of the as-prepared nanozymes. Bacterial suspensions treated with 64 μg mL^−1^ nanozyme exhibited significantly reduced optical density compared with untreated controls (Figure 3I), indicating inhibition of bacterial proliferation. Consistent with these observations, clear concentration-dependent inhibition zones were observed around disks containing PtPdCuFe NPs on agar plates (Figure 3J).

These findings demonstrate antibacterial activity of PtPdCuFe HEA nanozymes at 64 μg mL^−1^ and support their potential as a nanomaterial platform for combating bacterial infections [55]. Taken together, therefore, the overall antibacterial efficacy observed here reflects the combined contribution of the intrinsic POD-like activity exerted at physiological pH and the enhanced generation of intracellular reactive oxygen species occurring within locally acidified microenvironments. Nevertheless, how these two factors cooperate synergistically in vivo, particularly within infected tissues where complex biological barriers and dynamic metabolic conditions prevail, still requires further systematic investigation in future studies.

### 2.4. In Vitro Antibacterial Mechanism of PtPdCuFe HEA Nanozymes

**Figure 4:**
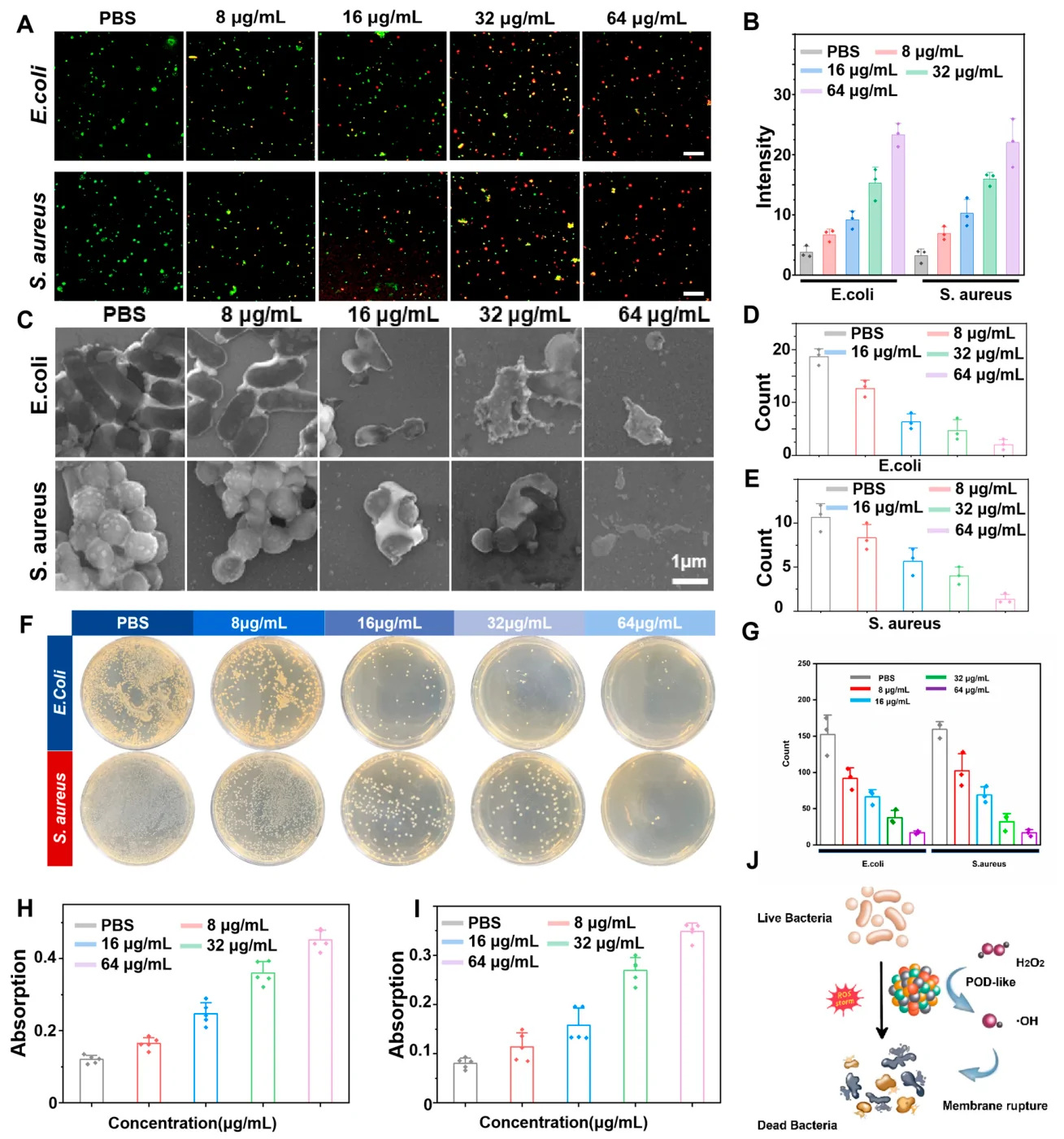
In vitro antibacterial mechanism of PtPdCuFe HEA nanozymes. (**A**) Live/Dead fluorescence images of *S. aureus* and *E. coli* treated with different concentrations of PtPdCuFe NPs. (**B**) Quantitative analysis of bacterial Live/Dead fluorescence intensity. (**C**) ESEM images of *S. aureus* and *E. coli* after treatment with different concentrations of PtPdCuFe NPs. (**D**) Quantitative analysis of membrane disruption in *E. coli*. (**E**) Quantitative analysis of membrane disruption in *S. aureus*. (**F**) Colony-counting assay of *S. aureus* and *E. coli* treated with different concentrations of PtPdCuFe NPs. (**G**) Quantitative bacterial survival analysis. (**H**) Extracellular protein leakage from *E. coli*. (**I**) Extracellular protein leakage from *S. aureus*. (**J**) Schematic illustration of the antibacterial mechanism of PtPdCuFe NPs. Experiments were repeated independently (**A**,**C**,**F**,**H**,**I**) three times with similar results.

To directly visualize the bactericidal effects of PtPdCuFe nanozymes against *E. coli* and *S. aureus*, bacterial Live/Dead dual-fluorescence staining followed by confocal microscopy was performed (Figure 4A). Green fluorescence represented viable bacteria with intact cell membranes, whereas red fluorescence indicated membrane-compromised dead bacteria. The PBS control group displayed predominantly green fluorescence with minimal red fluorescence. As the PtPdCuFe nanozyme concentration increased from 8 to 64 μg mL^−1^, both the number and fluorescence intensity of red-stained bacteria increased. Quantitative fluorescence analysis (Figure 4B) showed that, at 64 μg mL^−1^, the relative red fluorescence intensity was approximately sixfold higher for *E. coli* and sevenfold higher for *S. aureus* than in the PBS control. These results demonstrate that PtPdCuFe nanozymes induce severe bacterial membrane damage in a concentration-dependent manner. Combined with the enzyme-mimicking activity results described above, ROS generated through the POD-like activity of the nanozymes likely represent one of the major contributors to bacterial killing [56].

Environmental scanning electron microscopy (ESEM) was subsequently used to examine morphological alterations in *E. coli* and *S. aureus* following treatment with different concentrations of PtPdCuFe nanozymes (Figure 4C). PBS-treated *E. coli* exhibited regular short-rod morphology with smooth surfaces, whereas *S. aureus* cells showed an intact spherical morphology with continuous and intact membrane contours and no obvious wrinkling, collapse, or rupture. Following treatment with 16 μg mL^−1^ PtPdCuFe nanozymes, depressions and wrinkles appeared on the bacterial surfaces, accompanied by mild disruption of membrane integrity. At concentrations of 32 μg mL^−1^ or higher, extensive membrane rupture, edge dissolution, leakage of intracellular contents, and cellular collapse were observed in both bacterial species. Quantification of membrane-damaged bacteria (Figure 4D,E) showed a progressive increase in the proportion of damaged cells with increasing nanozyme concentration. At 64 μg mL^−1^, more than 90% of bacteria exhibited severe membrane disruption. These electron-microscopy observations suggest that ROS generated by the nanozyme preferentially attack the phospholipid bilayer of bacterial membranes and that destruction of this barrier contributes substantially to bacterial death.

The antibacterial activity of PtPdCuFe nanozymes against Gram-negative *E. coli* and Gram-positive *S. aureus* was quantified using colony-counting assays (Figure 4F,G). Numerous bacterial colonies were observed on PBS control plates, whereas the number of surviving colonies progressively decreased with increasing PtPdCuFe concentration. At 16 μg mL^−1^, the bactericidal efficiencies against *E. coli* and *S. aureus* were approximately 56% and 57%, respectively. At 64 μg mL^−1^, surviving colonies of both strains were markedly reduced, corresponding to approximately 90% killing. These findings demonstrate concentration-dependent, broad-spectrum antibacterial activity against both Gram-negative and Gram-positive bacteria. Because disruption of membrane integrity results in leakage of intracellular proteins, the extent of membrane damage was quantified by measuring extracellular protein levels (Figure 4H,I). Compared with the PBS control, extracellular protein levels increased progressively with increasing PtPdCuFe concentration in both *E. coli* and *S. aureus*. These results were consistent with the electron-microscopy and Live/Dead staining observations, indicating that the oxidative stress generated by the nanozymes continuously damages the bacterial cell membrane, increases membrane permeability, and promotes the release of intracellular metabolic enzymes and structural proteins. Consequently, bacteria lose the ability to maintain osmotic and metabolic homeostasis, ultimately resulting in irreversible cell death.

The proposed in vitro antibacterial mechanism of PtPdCuFe HEA nanozymes is summarized in Figure 4J. In the acidic microenvironment associated with bacterial wound infection, PtPdCuFe nanozymes exhibit POD-like catalytic activity and decompose H_2_O_2_ to generate strongly oxidizing •OH. These radicals attack the bacterial membrane, disrupt the phospholipid bilayer, increase membrane permeability, and induce extensive intracellular protein leakage. Subsequent destruction of the physiological barrier results in morphological collapse and loss of intracellular contents, ultimately producing broad-spectrum antibacterial effects against *E. coli* and *S. aureus*. These in vitro findings provide experimental support for the potential application of PtPdCuFe nanozymes in the treatment of bacteria-infected wounds [57,58,59,60].

### 2.5. Transcriptomic Analysis of the Antibacterial Effects of PtPdCuFe HEA Nanozymes

**Figure 5:**
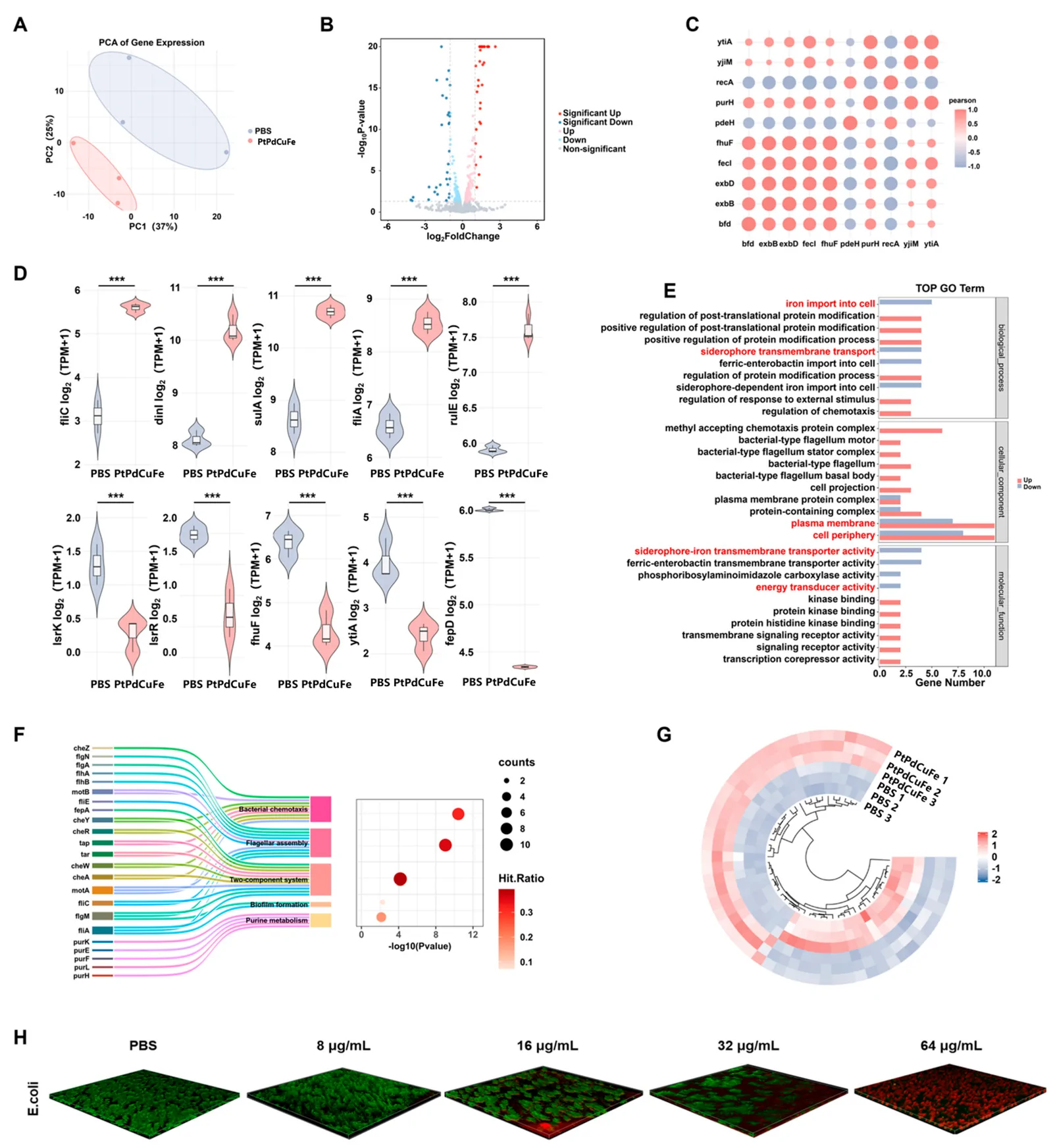
Transcriptomic analysis of the antibacterial effects of PtPdCuFe HEA nanozymes. (**A**) PCA showing clear separation between the control group (Ctrl, *n* = 3) and nanozyme-treated group (NPs, *n* = 3) along PC1 and PC2. PC1 and PC2 accounted for 37% and 25% of the total variance, respectively, corresponding to a cumulative explained variance of 62%. Ellipses represent 95% confidence intervals. (**B**) Volcano plot of DEGs. Red and blue points indicate significantly up-regulated (log_2_FC > 1, *p* < 0.05, *n* = 32) and down-regulated (log_2_FC < −1, *p* < 0.05, *n* = 22) genes, respectively; gray points represent genes without significant differential expression. (**C**) Pearson correlation heatmap of DEGs, showing the top 10 genes ranked by summed abundance. Red and blue indicate positive and negative correlations, respectively. (**D**) Expression distributions of the five most significantly up-regulated and five most significantly down-regulated genes in the Ctrl and NPs groups (*** represents *p* < 0.001, indicating extremely significant difference). Violin plots overlaid with box plots show the data distributions, medians, and quartiles. (**E**) GO enrichment analysis of DEGs (top 10 terms). (**F**) GSEA plots showing significant down-regulation of purine metabolism (NES = −1.63, *p* < 0.001) and siderophore transmembrane transport (NES = −1.62, *p* < 0.001) in the nanozyme-treated group. (**G**) A circular clustered heatmap. (**H**) Confocal Live/Dead images of *E. coli* membranes following treatment with different concentrations of PtPdCuFe NPs. Experiments were repeated independently three times with similar results.

To evaluate the global effects of nanozyme treatment on the bacterial transcriptome, principal component analysis (PCA) was first performed. As shown in Figure 5A, the control (Ctrl) and nanozyme-treated (NPs) groups were clearly separated along PC1 and PC2, indicating substantial transcriptomic remodeling following PtPdCuFe NP treatment and systematic differences in gene-expression profiles between the two groups. Differential expression analysis identified 54 significantly differentially expressed genes (DEGs), including 32 upregulated and 22 downregulated genes (Figure 5B). A circular clustered heatmap (Figure 5G) showed that the up-regulated and down-regulated genes formed two distinct clusters, indicating highly consistent expression patterns within each group. The five most strongly up-regulated genes were fliC (flagellin), sulA (cell-division inhibitor), dinI (SOS-response protein), fliA (flagellar sigma factor), and fliZ (flagellar regulator). The five most strongly down-regulated genes were lsrK (AI-2 kinase), fhuF (iron-associated reductase), lsrR (quorum-sensing regulator), fepD (iron transport), and bfd (iron-storage-associated protein) (Figure 5D). Among the down-regulated genes, extensive suppression of the iron-uptake system (fepA/D/G/C, exbB/D, and fhuF) and purine biosynthesis genes (purF/H/L/E/K) suggests that the bacteria underwent substantial remodeling of iron acquisition and energy metabolism in response to ROS stress. Conversely, stress-associated activation of flagellar/chemotaxis-related genes (fliC/A/Z and cheA/W/R/Y/Z), together with SOS-response genes such as recA and sulA, was consistent with perturbation of membrane-associated energy coupling and with DNA damage.

To investigate coordinated regulatory relationships among the DEGs, Pearson correlation coefficients were calculated using log_2_(TPM + 1) expression values for all 54 DEGs. The correlation heatmap (Figure 5C) showed strong positive correlations among up-regulated genes (r > 0.8) and among down-regulated genes, whereas strong negative correlations (r < −0.8) were observed between the two groups, consistent with the distinct transcriptional response patterns induced by PtPdCuFe NPs. Gene Ontology (GO) enrichment analysis (Figure 5E) was then used to examine the effects of nanozyme treatment on bacterial membrane-associated functions. Within the cellular component category, highly enriched terms included “plasma membrane,” “cell periphery,” and “plasma membrane protein complex,” indicating substantial perturbation of the bacterial membrane system. This interpretation was supported by crystal-violet staining (Figure S6) and confocal imaging of bacterial membranes (Figure 5H). Notably, strong enrichment of the flagellar stator complex and methyl-accepting chemotaxis protein (MCP) complex, with rich factors of 1.0 and 0.75, respectively, suggested marked disruption of proton-motive-force (PMF)-dependent energy-transduction systems at the transcriptional level. Among the biological-process terms, enrichment of iron-uptake-related processes, including siderophore transmembrane transport, reflected substantial remodeling of bacterial iron homeostasis. Enrichment of regulation-of-chemotaxis-related terms was consistent with a stress response to membrane damage, although dissipation of the PMF may impair effective chemotactic behavior. In the molecular-function category, enrichment of energy-transducer activity, together with down-regulation of exbB/exbD, supported disruption of membrane-associated proton-gradient-dependent energy transduction. Enrichment of siderophore–iron transmembrane transporter activity and kinase-binding terms suggested perturbations in iron homeostasis and intracellular signaling. KEGG pathway enrichment analysis (Figure S7) showed that PtPdCuFe NP treatment affected three major functional modules: signal sensing, including two-component systems; energy metabolism, including purine metabolism; and motility, including flagellar and chemotaxis pathways. Although motility-associated pathways were transcriptionally activated as part of the stress response, the loss of proton-motive force probably prevents this from translating into actual motility. In contrast, relatively limited alterations were observed in the biofilm-formation pathway. Gene set enrichment analysis (GSEA) was performed to investigate global alterations in key biological pathways. Purine metabolism and siderophore transmembrane transport were significantly down-regulated in the nanozyme-treated group (Figure 5F). The purine metabolism gene set contains genes encoding key enzymes involved in denovo purine biosynthesis (purF/H/L/E/K), and their coordinated down-regulation suggests substantial suppression of ATP/GTP biosynthesis. This effect is consistent with impaired energy coupling following disruption of the membrane proton gradient. The siderophore transmembrane transport pathway contains major components of the iron-uptake machinery, including fepA/D/G/C, exbB/D, and fhuF. Their coordinated down-regulation suggests suppression of iron uptake under ROS stress, potentially representing a bacterial response aimed at limiting iron-dependent oxidative reactions. Together, down-regulation of these pathways suggests that nanozyme treatment simultaneously perturbs membrane-associated energy metabolism and iron homeostasis, ultimately contributing to metabolic dysfunction and irreversible bacterial death.

Collectively, transcriptomic and functional enrichment analyses suggest that ROS generated by PtPdCuFe nanozymes initially damage the bacterial membrane system, leading to dissipation of the membrane proton gradient, perturbation of iron homeostasis, and suppression of energy metabolism. These combined effects ultimately disrupt ATP production and contribute to irreversible bacterial death.

## 3. Materials and Methods

### 3.1. Materials

Platinum(II) acetylacetonate (Pt(acac)_2_), palladium(II) acetylacetonate (Pd(acac)_2_), copper(II) acetylacetonate (Cu(acac)_2_), and iron(III) acetylacetonate (Fe(acac)_3_) were purchased from Sigma-Aldrich (St.Louis, MO, USA). Tungsten hexacarbonyl (W(CO)_6_), oleylamine (OAm), glucose, polyvinylpyrrolidone (PVP), 5,5-dimethyl-1-pyrroline N-oxide (DMPO), 2,2,6,6-tetramethylpiperidine (TEMP), sodium hypochlorite (NaClO), L-methionine, riboflavin, EDTA, and nitroblue tetrazolium chloride (NBT) were purchased from Macklin (Shanghai, China). DMEM, fetal bovine serum (FBS), trypsin–EDTA solution, Calcein-AM, propidium iodide (PI), DCFH-DA ROS probe, BCA protein assay kit, hydrogen peroxide assay kit, TMB assay kit, MB assay kit, and DMAO bacterial fluorescent dye were purchased from Beyotime (Shanghai, China). LB broth and LB agar were obtained from Solarbio (Beijing, China). The MTT assay kit was purchased from Beijing Coolaber Technology Co., Ltd. (Beijing, China). 

### 3.2. Synthesis of PtPdCuFe HEA Nanozymes

PVP (10 mg), glucose (24 mg), W(CO)_6_ (17.6 mg), Pt(acac)_2_ (4 mg), Pd(acac)_2_ (3 mg), Cu(acac)_2_ (2.6 mg), and Fe(acac)_3_ (3.6 mg) were dissolved in 10 mL of oleylamine in a Schlenk tube, followed by the addition of a borosilicate glass magnetic stirring bar. The mixture was ultrasonicated for 1 h to ensure homogeneous dispersion. The Schlenk tube was then purged with nitrogen three times. Under a nitrogen atmosphere, the tube was placed in a heating module and heated stepwise: the temperature was first raised to 50 °C, then to 100 °C, and subsequently to 150 °C. At this point, heating was temporarily paused; when the internal temperature reached 200 °C, the tube was cooled using a heat gun. Once the temperature dropped to 150 °C, the set temperature was raised to 200 °C. After the internal temperature reached 200 °C and continued to rise to 230 °C, the tube was again cooled with a heat gun. When the temperature decreased to 200 °C, the set temperature was adjusted to the final target of 230 °C, and the reaction was held at this temperature for 1 h. After the reaction was complete, the tube was allowed to cool naturally to room temperature. The obtained product was collected in a centrifuge tube, re-dispersed in 10 mL of cyclohexane under ultrasonication, and mixed with 30 mL of acetone (1:3, *v*/*v*). The nanoparticles were then centrifuged at 10,000× *g* for 30 min. This washing procedure was repeated three times, and the collected sample was dried and stored for further use [61].

For subsequent in vitro cell experiments, the nanoparticles were additionally washed twice with a petroleum ether/acetone mixture (1:40, *v*/*v*) to remove residual free oleylamine from the nanoparticle surfaces.

### 3.3. Material Characterization

X-ray diffraction. Samples for XRD analysis were prepared using a thin-film method. PtPdCuFe nanoparticles were dispersed in cyclohexane to obtain a concentrated suspension, which was drop-cast onto clean fluorine-doped tin oxide (FTO) glass and thoroughly dried. XRD patterns were collected using a D8 ADVANCE diffractometer (Bruker AXS, Karlsruhe, Germany). Phase identification was performed using Jade 9.0, and the diffraction peaks were compared with standard PDF cards for Pt, Pd, Cu, and Fe.

Transmission electron microscopy. PtPdCuFe nanoparticle suspensions were appropriately diluted and drop-cast onto carbon-film-coated molybdenum grids (molybdenum was used in place of copper to avoid interference with the Cu signal in EDS analysis), followed by drying at room temperature. Nanoparticle morphology was characterized using a HT7820 TEM (Hitachi High-Tech, Tokyo, Japan) operated at an accelerating voltage of 100 kV. Experiments were repeated independently three times with similar results.

Aberration-corrected scanning transmission electron microscopy. Atomic-resolution HAADF-STEM images were obtained using an Talos F200X G2 transmission electron microscope (Thermo Fisher Scientific, Waltham, MA, USA) operated at 200 kV. GPA was used to analyze local strain distributions in selected representative nanoparticles. Experiments were repeated independently three times with similar results.

ICP-MS. Samples were digested using aqua regia. After calibration with standard curves, the contents of Pt, Pd, Cu, and Fe were determined using an 7700x ICP-MS (Agilent Technologies, Santa Clara, CA, USA).

### 3.4. Evaluation of Enzyme-Mimicking Activities and Particle Stability

POD-like activity. TMB was used as a chromogenic substrate to evaluate the POD-like activity of PtPdCuFe NPs. The total reaction volume was 1.0 mL and contained acetate buffer (pH 5.5), PtPdCuFe NPs (64 μg mL^−1^), TMB (0.5 mM), and H_2_O_2_ (10 mM). After incubation at 37 °C for 10 min, UV–Vis absorption spectra from 400 to 800 nm were collected using an Evolution 350 UV–Vis spectrophotometer (Thermo Fisher Scientific, Waltham, MA, USA). Control groups comprised buffer alone, H_2_O_2_ alone, and PtPdCuFe NPs alone; PtPdCuFe NPs + H_2_O_2_ was the complete reaction. The final TMB concentration was maintained at 0.5 mM in all groups. Each experiment was independently repeated at least three times. Experiments were repeated independently three times with similar results.

Methylene blue degradation assay. The oxidative catalytic activity of PtPdCuFe NPs was evaluated using MB degradation [62]. The reaction mixture consisted of acetate buffer (pH 5.5), MB (10 μg mL^−1^), PtPdCuFe NPs (64 μg mL^−1^), and H_2_O_2_ (10 mM) and was incubated at 37 °C for 15 min. UV–Vis spectra were collected from 400 to 800 nm. After correction for light-scattering background caused by the nanoparticles, MB degradation was evaluated based on the decrease in absorbance at 664 nm. Control groups consisted of buffer alone, H_2_O_2_ alone, and PtPdCuFe NPs alone. Each experiment was independently repeated three times.

EPR detection of hydroxyl radicals. DMPO was used as a spin-trapping probe for the EPR detection of •OH. The total reaction volume was 300 μL and contained acetate buffer (pH 5.5), PtPdCuFe NPs (64 μg mL^−1^), H_2_O_2_ (10 mM), and DMPO (100 mM). EPR spectra were acquired using an EPR200-Plus spectrometer (CIQTEK, Hefei, China). Experiments were repeated independently three times with similar results.

Dissolved oxygen measurement. CAT-like activity was evaluated by monitoring O_2_ production during PtPdCuFe NP-mediated decomposition of H_2_O_2_. A sealed reaction vessel containing PBS (pH 7.4) and 1 mM H_2_O_2_ was used, with a total reaction volume of 1 mL. Once the baseline dissolved-oxygen signal stabilized, PtPdCuFe NPs were added to a final concentration of 64 μg mL^−1^. The reaction mixture was maintained at 37 °C and continuously stirred at 300 rpm. Dissolved oxygen was monitored for 300 s using an Oxi 3315 dissolved-oxygen electrode (WTW, Weilheim, Germany). PBS containing H_2_O_2_ without nanoparticles served as the control. Each experiment was independently repeated three times. Experiments were repeated independently three times with similar results.

H_2_O_2_ consumption assay. PtPdCuFe NPs (64 μg mL^−1^) were incubated with 1 mM H_2_O_2_ in PBS at pH 7.4 and 37 °C. Samples were collected at 0, 5, 10, 15, 20, and 25 min. The remaining H_2_O_2_ was quantified using an H_2_O_2_ assay kit according to the manufacturer’s instructions. Absorbance was measured at 550 nm using a Synergy H1 microplate reader (BioTek Instruments, Winooski, VT, USA). Matrix-matched standard curves were prepared in parallel for each experiment. After subtraction of blank values and nanoparticle-derived absorbance, the remaining H_2_O_2_ content was normalized to the value at 0 min. Experiments were independently repeated three times. Experiments were repeated independently three times with similar results.

EPR evaluation of singlet-oxygen scavenging. TEMP was used as a spin-trapping probe to assess ^1^O_2_-scavenging activity. An H_2_O_2_ + NaClO singlet-oxygen-generating system was prepared in PBS at pH 7.4. TEMP was added to a final concentration of 100 mM, followed by PtPdCuFe NPs at a final concentration of 64 μg mL^−1^, after which EPR spectra were collected [63]. Experiments were repeated independently three times with similar results.

NBT photoreduction assay for SOD-like activity. SOD-like activity was evaluated using an NBT photoreduction assay. The reaction system consisted of methionine (13 mM), NBT (75 μM), EDTA (100 μM), riboflavin (20 μM), and PtPdCuFe NPs (20 μg mL^−1^) in phosphate buffer at pH 7.4. The reaction was initiated by illumination at 4000 lux for 20 min, followed by measurement of absorbance at 560 nm using a microplate reader (Molecular Devices, LLC, San Jose, CA, USA). SOD-like activity was calculated from the percentage inhibition of NBT photoreduction. Each experiment was independently repeated three times.

Colloidal and catalytic stability. PtPdCuFe nanoparticles were dispersed in PBS and maintained at 25 °C. Their PDI values were measured at 0, 6, 12, and 24 h using a Zetasizer Nano ZS90 (Malvern Panalytical, Malvern, UK) to evaluate colloidal stability. For catalytic stability, PtPdCuFe NPs were stored at pH 5.5 or pH 7.4 and sampled after 12, 24, 36, and 48 h. POD-like activity was reassessed using the TMB assay at pH 5.5, whereas CAT- and SOD-like activities were evaluated at pH 7.4 using the corresponding methods described above. Experiments were repeated independently three times with similar results.

### 3.5. In Vitro Biocompatibility

Cell culture. THLE-2 cells were purchased from Procell (Wuhan Pricella Biotechnology Co., Ltd., Wuhan, China; catalog no. CL-0833) and cultured in DMEM supplemented with 10% FBS at 37 °C in a humidified incubator containing 5% CO_2_.

Live/Dead cell staining. Cells were seeded into 24-well plates at a density of 5 × 10^4^ cells per well and allowed to adhere overnight. The medium was replaced with fresh medium containing PtPdCuFe NPs at concentrations of 0, 8, 16, 32, or 64 μg mL^−1^, followed by incubation for 24 h. Cells were washed three times with PBS and stained with Calcein-AM and PI for 15 min in the dark. Fluorescence images were acquired using an inverted laser-scanning confocal microscope (TCS SP8; Leica Microsystems, Wetzlar, Germany). At least five randomly selected, non-overlapping fields were collected for each sample. A consistent segmentation threshold was applied to all groups. Cell viability was calculated as the number of Calcein-AM-positive cells divided by the total number of stained cells. Three independent experiments were performed.

Cytotoxicity assay. THLE-2 cells were seeded into 96-well plates at 5 × 10^3^ cells per well and allowed to adhere overnight. Cells were treated with PtPdCuFe NPs at concentrations of 0, 8, 16, 32, or 64 μg mL^−1^ for 24 h. Subsequently, 10 μL of MTT solution was added to each well containing 90 μL of culture medium, and the plates were incubated at 37 °C for 2 h. The medium was then removed and the formazan crystals were dissolved in 100 μL of DMSO with gentle shaking. Absorbance at 490 nm was measured using a Synergy H1 microplate reader (BioTek, Winooski, VT, USA). Three independent experiments were conducted.

Hemolysis assay. Commercial mouse red blood cells (10% suspension, Cat. No. SBJ-RBC-M005, Sbjbio, Nanjing, China) were used in this study. Erythrocytes were repeatedly washed with PBS until the supernatant became colorless. A 2% (*v*/*v*) erythrocyte suspension was then prepared. Equal volumes of erythrocyte suspension and nanoparticle dispersions were mixed to obtain final PtPdCuFe NP concentrations of 8, 16, 32, and 64 μg mL^−1^. PBS and ultrapure water were used as the negative and positive controls, respectively. After incubation at 37 °C for 2 h, the samples were centrifuged at 1500× *g* for 10 min, and the absorbance of the supernatant at 540 nm was measured and corrected for nanoparticle background absorbance. Experiments were repeated independently three times with similar results.

### 3.6. In Vitro Antibacterial Activity and Mechanistic Evaluation

Bacterial strains and treatment. Gram-negative *E. coli* (ATCC 25922) and Gram-positive *S. aureus* (ATCC 25923) were used as model bacterial strains. Bacteria were cultured overnight in LB broth at 37 °C with shaking at 180 rpm. Bacteria in the logarithmic growth phase were collected and diluted with sterile PBS to approximately 1 × 10^6^ CFU mL^−1^. Bacterial suspensions were incubated in PBS at pH 5.5 for 4 h at 37 °C in the presence of PtPdCuFe NPs at final concentrations of 0, 8, 16, 32, or 64 μg mL^−1^ and H_2_O_2_ at a final concentration of 100 μM. Control groups comprised PBS alone, H_2_O_2_ alone, and PtPdCuFe NPs alone. The solvent volume was maintained constant across all groups. Each experiment was independently repeated at least three times.

Colony-counting assay. Following treatment, the bacterial suspensions were serially diluted with sterile PBS. An aliquot of 100 μL from an appropriate dilution was spread onto LB agar plates and incubated at 37 °C for 24 h. Plates containing 30–300 colonies were selected for colony counting.

Bacterial viability assay. *E. coli* and *S. aureus* were separately inoculated into 96-well plates at approximately 1 × 10^6^ CFU mL^−1^ and incubated overnight with shaking. Bacteria were treated with PtPdCuFe NPs at 0, 8, 16, 32, or 64 μg mL^−1^ for 16 h. Subsequently, 10 μL of MTT solution was added to each well containing 90 μL of culture medium, and the plates were incubated at 37 °C for 4 h. Absorbance at 490 nm was measured using a Synergy H1 microplate reader. Three independent experiments were performed.

Bacterial Live/Dead confocal imaging. After 4 h of treatment, bacteria were collected by centrifugation at 8000× *g* for 5 min, washed twice with sterile PBS, and resuspended. The bacteria were stained with DMAO and PI for 15 min in the dark. Images were subsequently acquired using an inverted laser-scanning confocal microscope (TCS SP8; Leica Microsystems, Wetzlar, Germany). At least five random fields were collected for each sample.

Detection of intracellular bacterial ROS. Bacteria treated with different concentrations of PtPdCuFe NPs were collected and washed twice with PBS. DCFH-DA was added at a final concentration of 10 μM, and the bacteria were incubated at 37 °C for 30 min in the dark. Unbound probe was removed by PBS washing, and intracellular fluorescence was analyzed using a BD FACSAria II flow cytometer with excitation and emission wavelengths of 488 and 530 nm, respectively.

Bacterial growth turbidity assay. Bacterial suspensions at approximately 1 × 10^6^ CFU mL^−1^ were inoculated into LB broth containing PtPdCuFe NPs at concentrations of 0, 8, 16, 32, or 64 μg mL^−1^. Cultures were incubated at 37 °C with shaking at 180 rpm for 24 h. Bacterial growth was evaluated by measuring the optical density at 550 nm using a microplate reader. Three independent experiments were performed.

Agar disk-diffusion assay. Bacterial suspensions were adjusted to a 0.5 McFarland standard (approximately 1 × 10^8^ CFU mL^−1^) and uniformly spread onto LB agar plates. Sterile 6-mm filter-paper disks were placed on the agar surface, and 20 μL of PtPdCuFe NP dispersion at 64 μg mL^−1^ was applied to each disk. Plates were incubated at 37 °C for 24 h. Three biological replicates were performed.

Scanning electron microscopy. *E. coli* and *S. aureus* suspensions were adjusted to approximately 1 × 10^7^ CFU mL^−1^ and treated with different concentrations of PtPdCuFe NPs for 4 h. Bacterial cells were collected by centrifugation, washed with PBS, and fixed with 2.5% glutaraldehyde at 4 °C for 1 h. Samples were sequentially dehydrated using 30%, 50%, 70%, 90%, and 100% ethanol, subjected to critical-point drying, and sputter-coated with gold for 60 s. Bacterial morphology was observed using a scanning electron microscope (Quattro S, Thermo Fisher Scientific, Waltham, MA, USA) at an accelerating voltage of 10 kV.

Quantification of extracellular protein leakage. Following bacterial treatment, the suspensions were centrifuged at 8000× *g* for 10 min, and the supernatants were collected. Extracellular protein leakage was quantified using a BCA protein assay kit according to the manufacturer’s instructions. Experiments were repeated independently three times with similar results.

Confocal imaging of bacterial biofilms. Overnight cultures of *E. coli* were diluted to approximately 1 × 10^7^ CFU mL^−1^. Sterile coverslips were placed in 24-well plates, and bacterial suspensions containing different concentrations of PtPdCuFe NPs were added. Biofilms were formed by static incubation at 37 °C for 24 h. Coverslips were washed three times with PBS and stained with DMAO and PI for 15 min in the dark. Z-stack images were acquired using an inverted laser-scanning confocal microscope with a *Z*-axis step size of 0.5 μm. Three independent experiments were conducted.

Crystal-violet staining of bacterial biofilms. *E. coli* suspensions at approximately 1 × 10^7^ CFU mL^−1^ were seeded into flat-bottom 96-well plates and treated with different concentrations of PtPdCuFe NPs. Biofilms were allowed to develop by static incubation at 37 °C for 24 h. Planktonic bacteria were removed, and the wells were washed three times with PBS. Biofilms were fixed with methanol for 15 min and stained with 0.1% crystal violet for 15 min. Excess dye was removed using ultrapure water, and the plates were dried at room temperature. The bound dye was solubilized in 33% acetic acid and quantified by absorbance at 590 nm. At least six technical replicates were included per group, and the complete experiment was independently repeated three times.

Bacterial transcriptome sequencing and analysis. Transcriptome sequencing was performed by Sangon Biotech (Shanghai) Co., Ltd. (Shanghai, China). *E. coli* samples were divided into a control group (Ctrl, *n* = 3) and a PtPdCuFe NP-treated group (NPs, *n* = 3). Total RNA was extracted using a Total RNA Extractor kit (TRIzol; Sangon Biotech, catalog no. B511311) according to the manufacturer’s instructions. RNA concentration and purity were determined using a Qubit 2.0 fluorometer (Invitrogen, Thermo Fisher Scientific, Waltham, MA, USA) and an SMA4000 microspectrophotometer (Merinton Instrument, Beijing, China), respectively. RNA integrity and potential genomic DNA contamination were evaluated by agarose-gel electrophoresis. All samples met the required quality criteria and were used for subsequent library construction. Raw sequencing reads were assessed using FastQC (version 0.11.9). Filtered clean reads were aligned to the *E. coli* reference genome, and gene-level read counts were obtained. Differential gene-expression analysis was performed using the DESeq2 package (version 1.44.0) in R version 4.4.1. DEGs were defined as those with |log_2_ fold change| > 1 and a Benjamini–Hochberg-adjusted *p* value (padj) < 0.05. PCA and hierarchical clustering were performed to evaluate sample reproducibility and global transcriptional patterns. Pearson correlation coefficients were calculated based on log_2_(TPM + 1) values. GO and KEGG enrichment analyses were conducted for the DEGs, with *p* < 0.05 considered statistically significant. GSEA was performed using the desktop version of GSEA software (version 4.1.0) with 1000 gene-set permutations. Unless otherwise specified, all bioinformatics analyses were conducted in R.

### 3.7. Statistical Analysis

All experiments were performed in triplicate. Data are displayed as mean ± standard deviation (SD) at least in triplicate (in the form of Box or Violin plots). One-way analysis of variance (ANOVA) was used to examine the differences among three or more groups when the residues conform to a Gaussian distribution, and the comparison for multiple comparisons was affirmed by Tukey’s post-hoc test. For certain data exhibiting a non-Gaussian distribution, their statistical information was analyzed using Kruskal–Wallis’s test.

### 3.8. Graphical Abstract

The graphical abstract was generated with the assistance of ChatGPT 6.1. All content was manually reviewed, revised and approved by the authors. We confirm that there are no copyright issues.

## 4. Conclusions

In this study, a PtPdCuFe-HEA nanozyme with multiple enzyme-mimicking activities and microenvironment-responsive catalytic behavior was developed to address the complex pathological conditions of bacteria-infected wounds, in which bacterial colonization, oxidative stress, and tissue hypoxia coexist. Ultrasmall nanoparticles with an average diameter of 2.14 ± 0.18 nm, excellent monodispersity, and good colloidal stability were synthesized with good shape control via an oleylamine-assisted pyrolysis method. Aberration-corrected electron microscopy, EPR spectroscopy, and transcriptomic analysis were employed to investigate the microscopic structure, catalytic behavior, and antibacterial mechanism of the nanozyme. Defects such as fivefold twins, twin boundaries, grain boundaries, and locally ordered domains may provide abundant catalytic active sites for the as-prepared nanozyme, while synergistic interactions among the constituent metals may account for the pH-gated POD-, CAT-, and SOD-like activities of the PtPdCuFe nanozyme.

Under acidic conditions, the nanozyme decomposes H_2_O_2_ into •OH; under neutral conditions, approximately 84% of H_2_O_2_ is removed within 25 min, and the O_2_ concentration increases to approximately 50% within 60 s. The catalytic activity remains stable for up to 48 h. At 16 µg·mL^−1^, the bactericidal efficiencies against *E. coli* and *S. aureus* are approximately 56% and 57%, respectively; at 64 µg·mL^−1^, killing reaches approximately 90%, and more than 90% of bacteria exhibit severe membrane disruption. Transcriptomic analysis indicates that nanozyme treatment disrupts the bacterial membrane proton gradient, disturbs ion homeostasis and purine metabolism, and ultimately impairs bacterial energy metabolism. At 64 µg·mL^−1^, approximately 90% of mammalian cells remain viable and the hemolysis rate stays below 5%, indicating good biocompatibility. This study provides preliminary evidence for a structure–catalysis relationship and proposes the hypothesis that defect structures govern multi-enzyme catalytic activity, thereby identifying PtPdCuFe-HEA nanozymes as a candidate antibacterial material for bacteria-infected wounds.

Further animal studies are required to systematically evaluate their in vivo metabolic behavior, long-term safety, and effects on wound repair [63,64,65,66,67,68].

## Data Availability

The data presented in this study are available on request from the corresponding author.

## Supplementary Materials

The following supporting information can be downloaded at https://www.mdpi.com/article/10.3390/molecules31193506/s1: Figure S1: Radial intensity profile obtained by integrating the selected-area electron diffraction (SAED) ring patterns. Figure S2: Interplanar-spacing measurement of the first particle type. Figure S3: Interplanar-spacing measurement of the second particle type. Figure S4: Interplanar-spacing measurement of the third particle type. Figure S5: biological process (BP). Figure S6: Crystal violet staining under treatment with different concentrations of nanoparticles. Figure S7: GSEA plots showing significant downregulation of purine metabolism (NES = −1.63, *p* < 0.001) and siderophore transmembrane transport (NES = −1.62, *p* < 0.001) in the nanozyme-treated group. Figure S8: Lollipop plot for three Gene Ontology (GO) categories: molecular function (MF), and cellular component (CC). Figure S9: Synthetic-process screening of PtPdCuFe HEA nanozymes. Figure S10: Contour plots of stress–strain distributions in the remaining directions for three typical PtPdCuFe HEA nanozymes. Figure S11: Experimental apparatus.
