# Supplementary material for: Antibacterial Performance of Defect-Enriched PtPdCuFe High-Entropy Alloy Nanozymes
Source: Molecules. 2026 Oct 1;31(19):3506. doi: 10.3390/molecules31193506 (PMC13666288; doi:10.3390/molecules31193506)
Supplement: Supplementary file 1 [file molecules-31-03506-s001.zip › molecules-4604961-supplementary.pdf]

Supplementary Material
Antibacterial Performance of Defect-Enrich PtPdCuFe High-Entropy Alloy Nanozymes

Jiawei Zhang [1], Zhiyuan Li [1], Weng-Chon (Max) Cheong [1,*], Awanisa Wubuli [2], Sitraka Ny Aina Raharivelo [3] and Ziheng Chen [4]

[1] Macao Institute of Materials Science and Engineering (MIMSE), Macau University of Science and Technology, Macao SAR 999078, China

[2] School of Chinese Materia Medica, China Pharmaceutical University, Nanjing, Jiangsu, 211198, China

[3] State Key Laboratory of Climate System Prediction and Risk Management/Key Laboratory of Meteorological Disaster, Ministry of Education/Collaborative Innovation Center on Forecast and Evaluation of Meteorological Disasters, Nanjing University of Information Science and Technology, Nanjing, Jiangsu, 210044, China; 202451010005@nuist.edu.cn

[4] School of Civil Engineering and Architecture, Anhui University of Science & Technology, Huainan, Anhui, 232001, China

* Correspondence: wccheong@must.edu.mo

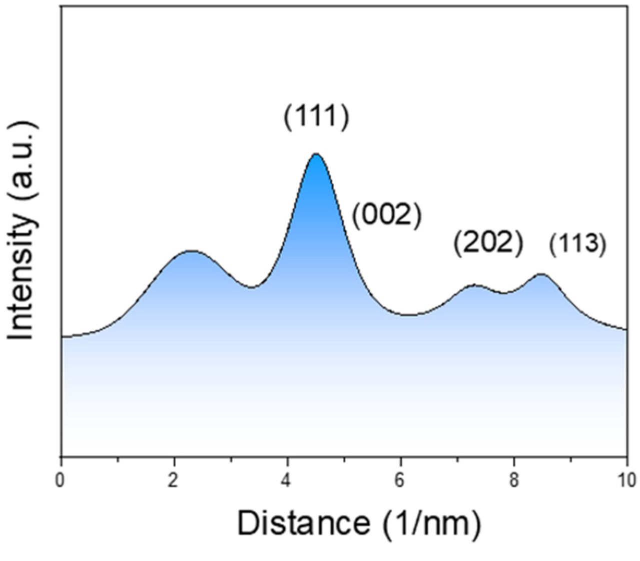

Figure S1. Radial intensity profile obtained by integrating the selected-area electron diffraction (SAED) ring patterns.

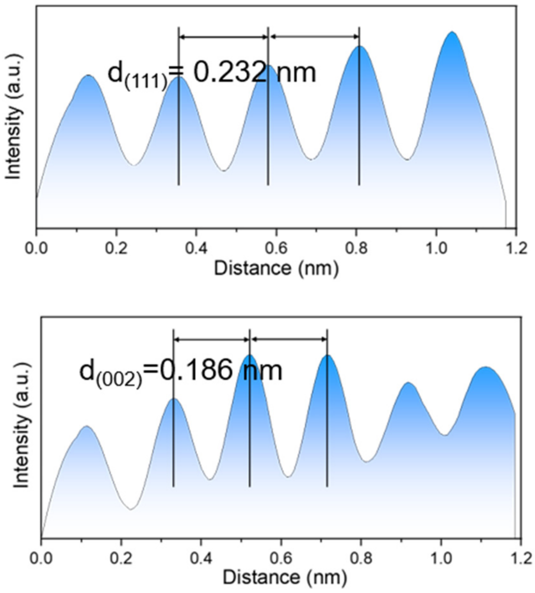

Figure S2. Interplanar-spacing measurement of the first particle type.

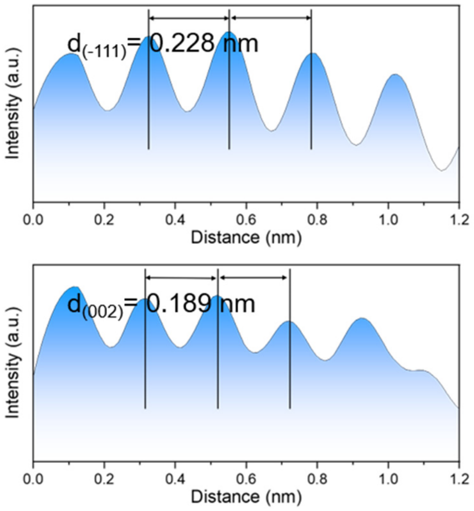

Figure S3. Interplanar-spacing measurement of the second particle type.

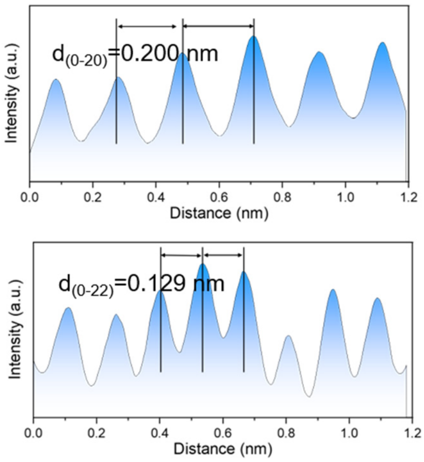

Figure S4. Interplanar-spacing measurement of the third particle type.

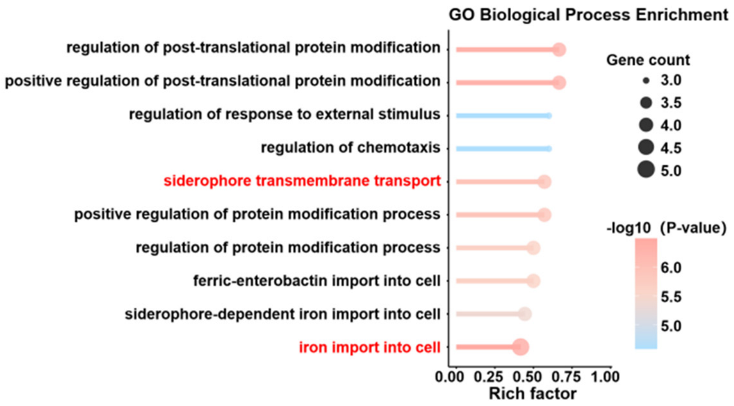

Figure S5. biological process (BP).

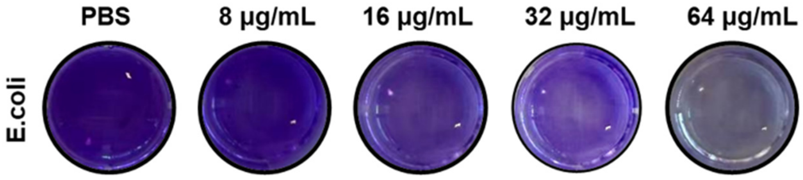

Figure S6. Crystal violet staining under treatment with different concentrations of nanoparticles.

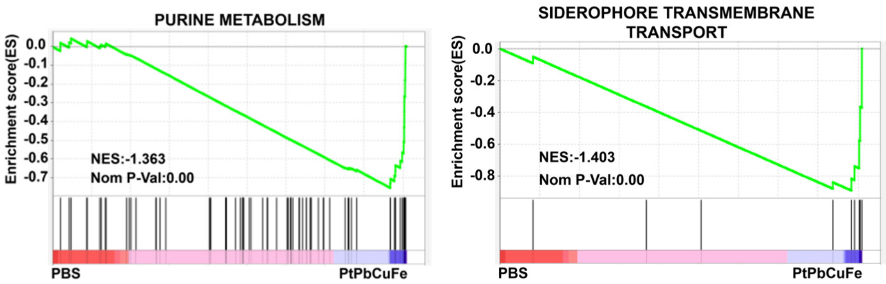

Figure S7. GSEA plots showing significant downregulation of purine metabolism (NES = −1.63, P < 0.001) and siderophore transmembrane transport (NES = −1.62, P < 0.001) in the nanozyme-treated group.

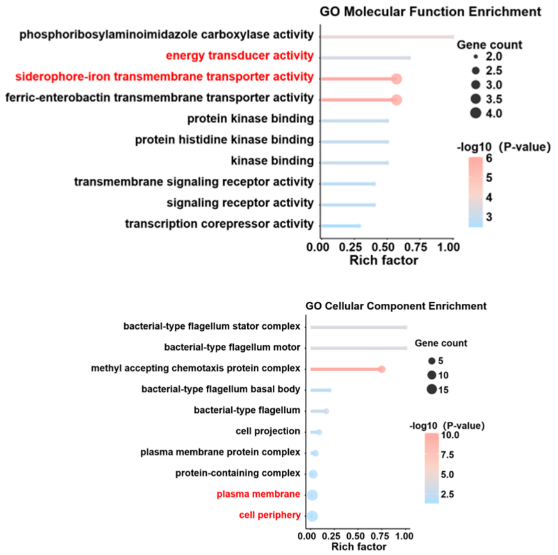

Figure S8. Lollipop plot for three Gene Ontology (GO) categories: molecular function (MF), and cellular component (CC).

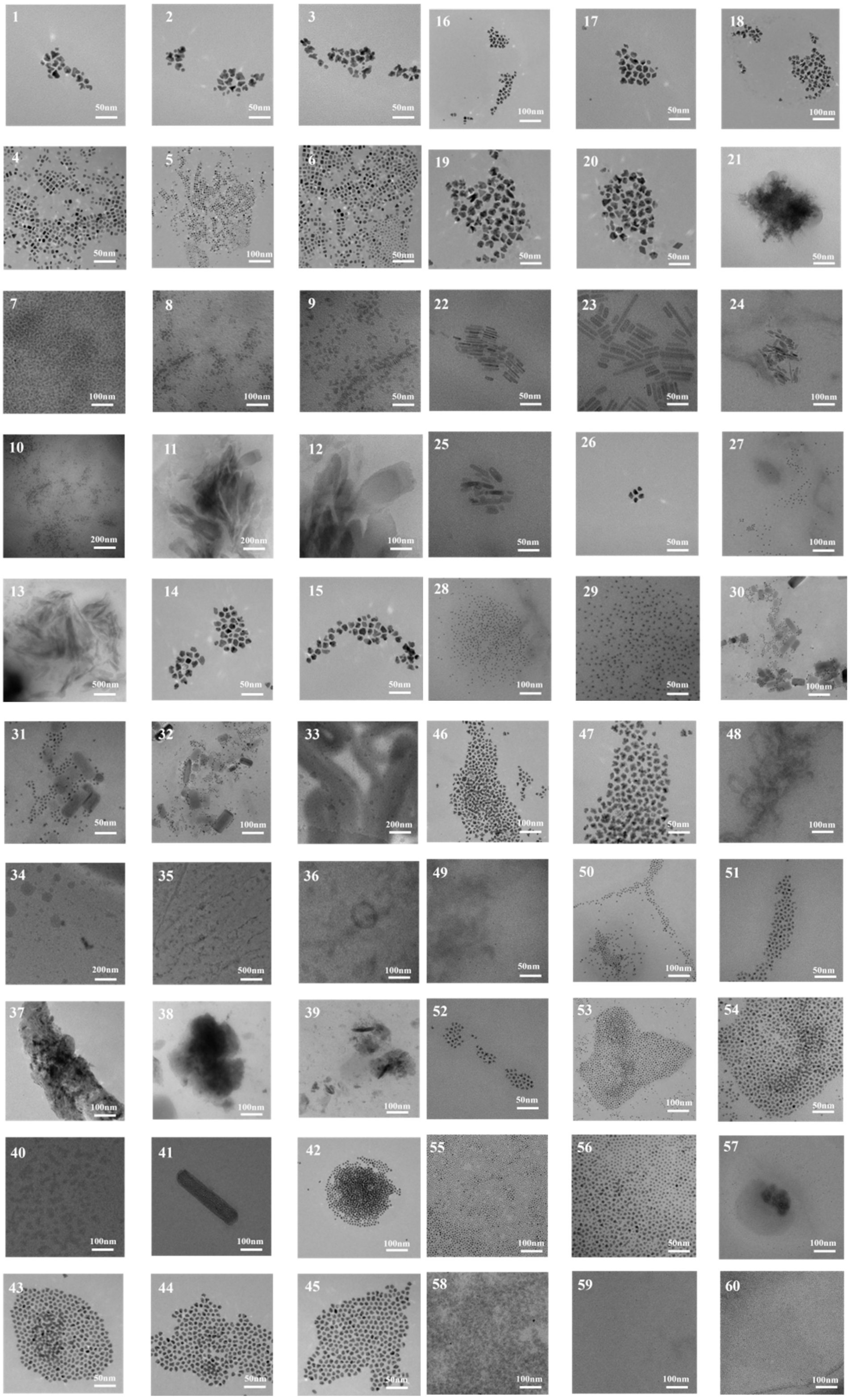

Figure S9. Synthetic-process screening of PtPdCuFe HEA nanozymes.

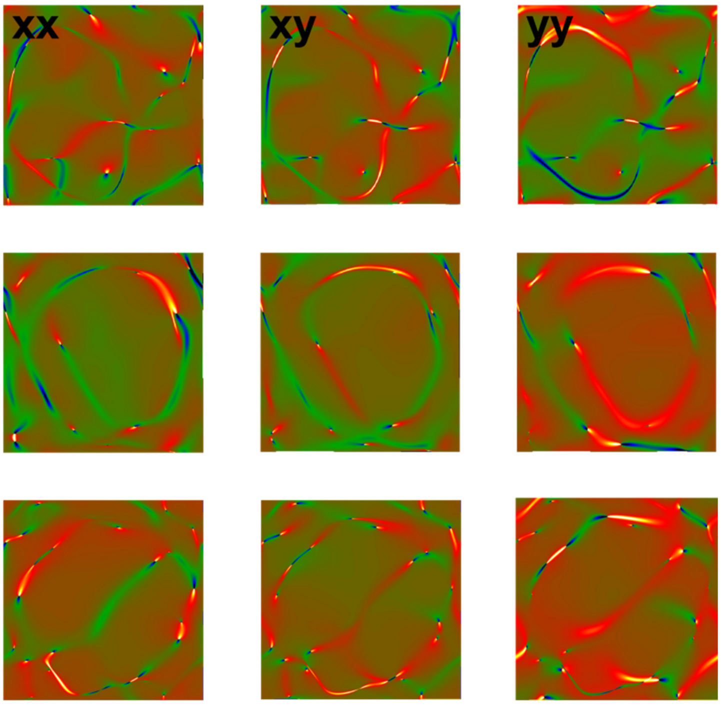

Figure S10. Contour plots of stress-strain distributions in the remaining directions for three typical PtPdCuFe HEA nanozymes.

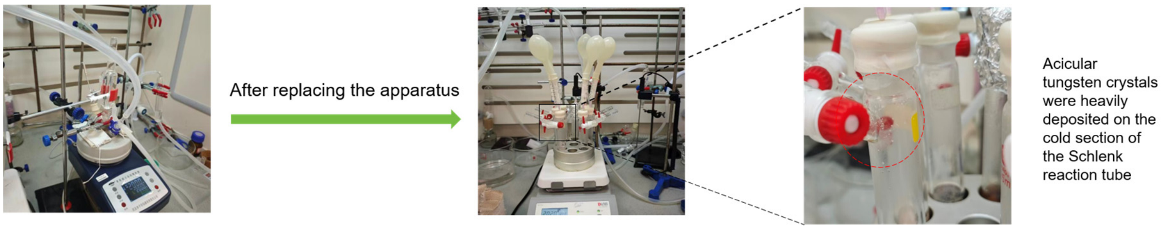

Figure S11. Experimental apparatus.
